# Norwegian scabies coinfected with disseminated *Talaromyces marneffei* – a dual challenge in HIV immunodeficiency: A case report

**DOI:** 10.1097/MD.0000000000043792

**Published:** 2025-08-08

**Authors:** Yan Zhang, Xiaojin Ding, Jing Wu, Wenyan Yu, Kenv Pan, Aifang Xu

**Affiliations:** a Department of Clinical Laboratory, Hangzhou Xixi Hospital, Hangzhou Sixth People’s Hospital, Hangzhou Xixi Hospital Affiliated to Zhejiang Chinese Medical University, Hangzhou, Zhejiang Province, China; b Department of Infectious Diseases Department II, Hangzhou Xixi Hospital, Hangzhou Sixth People’s Hospital, Hangzhou Xixi Hospital Affiliated to Zhejiang Chinese Medical University, Hangzhou, Zhejiang Province, China.

**Keywords:** HIV, Norwegian scabies, *Talaromyces marneffei*

## Abstract

**Rationale:**

Norwegian scabies (crusted scabies) is an extreme manifestation of *Sarcoptes scabiei* infestation, primarily affecting immunocompromised populations such as HIV-positive individuals. This condition is characterized by a severe mite burden and high transmissibility, often accompanied by pathognomonic hyperkeratotic plaques.

**Patient concerns:**

A 46-year-old male patient diagnosed with HIV presented with persistent fever and progressive cutaneous desquamation, along with lymphadenopathy and worsening rash, initially misdiagnosed as atopic dermatitis.

**Diagnoses:**

Microscopic examination of skin scrapings confirmed a diagnosis of Norwegian scabies. Blood cultures and lymph node puncture revealed disseminated *Talaromyces marneffei* infection. The patient’s cluster of differentiation 4 cell count was extremely low, and immunoglobulin E levels were significantly elevated.

**Interventions:**

The patient received a comprehensive treatment regimen including topical sulfur ointment, oral ivermectin, and voriconazole, in addition to antiretroviral therapy. Supportive care included methylprednisolone and immunoglobulin therapy.

**Outcomes:**

After 1 month of treatment, the patient’s skin symptoms completely resolved, and he was discharged.

**Lessons:**

For immunocompromised HIV patients, routine microscopic examination of skin scrapings is recommended to promptly identify Norwegian scabies, preventing misdiagnosis and potential infectious complications.

## 1. Introduction

Norwegian scabies (crusted scabies) represents an extreme manifestation of *Sarcoptes scabiei* infestation, distinguished by an overwhelming mite burden, profound transmissibility, and pathognomonic hyperkeratotic plaques. Initially identified in Norwegian leprosy patients, this condition predominantly afflicts immunocompromised populations, including HIV-positive individuals, immunosuppressive therapy recipients, and geriatric patients with neuropsychiatric disorders or impaired sensorium.^[1,2]^ The classic presentation features generalized erythema, desquamation, and pronounced crust formation; however, HIV-associated cases frequently manifest as non-pruritic, verrucous plaques that mimic psoriasis, atopic dermatitis, eczema, or drug-induced eruptions.^[3]^ Without adequate treatment, secondary infections can develop leading to bacterial sepsis with historically high fatality.^[1]^ *Talaromyces marneffei* (TM, formerly *Penicillium marneffei*), a thermally dimorphic opportunistic fungus, primarily targets immunodeficient hosts, typically presenting with papulopustular lesions accompanied by constitutional symptoms such as pyrexia and lymphadenopathy.^[4]^ Reports of scabies infections among newly diagnosed HIV patients are uncommon, and instances of coinfection with TM are even rarer. This case report details a 46-year-old male patient recently diagnosed with HIV and TM infection, who was subsequently diagnosed with Norwegian scabies following a microscopic examination of skin scrapings that confirmed the presence of scabies mites. The patient received treatment and was discharged within 1 month.

## 2. Case presentation

**Figure 1.**
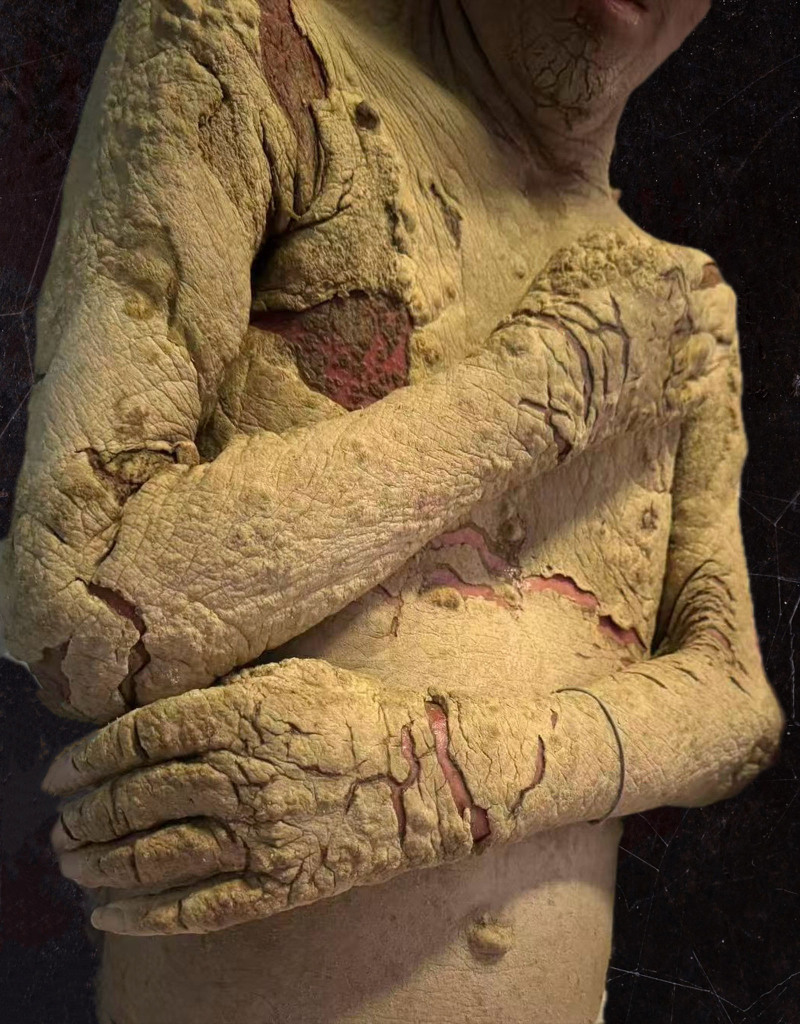
Clinical presentation of Norwegian scabies: extensive hyperkeratotic crusting resembling oyster shells, widespread desquamation, and numerous dark red macules and patches distributed across the body.

**Figure 2.**
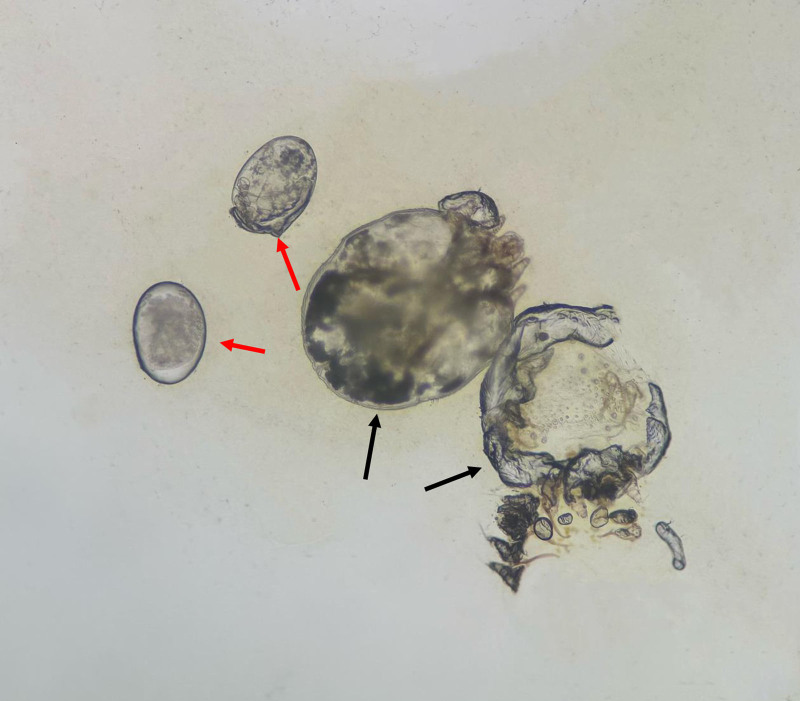
Laboratory findings under the microscope (1000×): scabiei mite (black arrow), eggs (red arrow).

**Figure 3.**
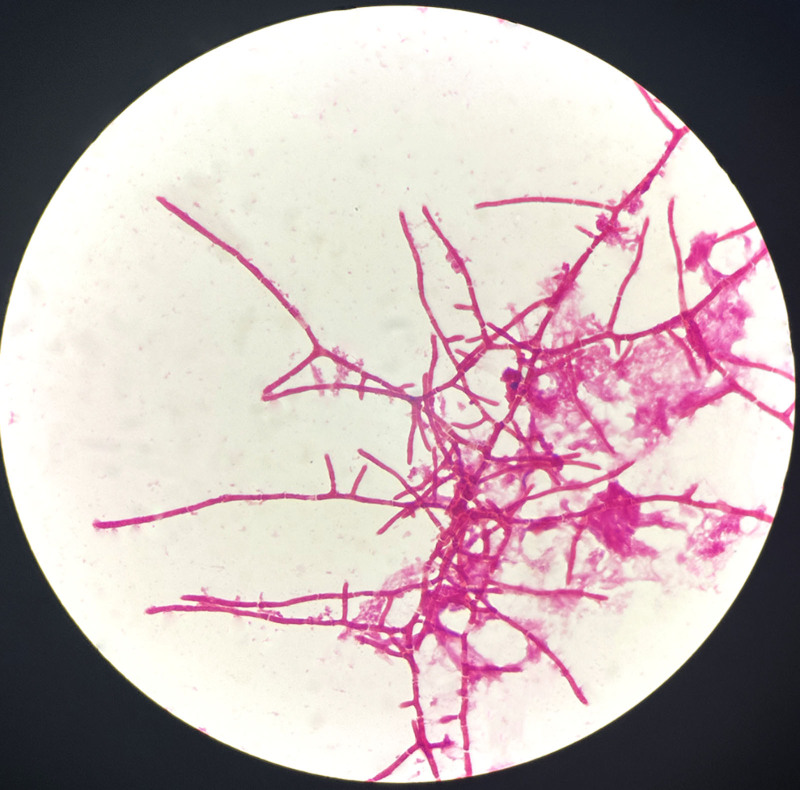
Laboratory findings under the microscope (1000×): gram staining showed numerous yeast-like organisms after a 24 h blood culture.

**Figure 4.**
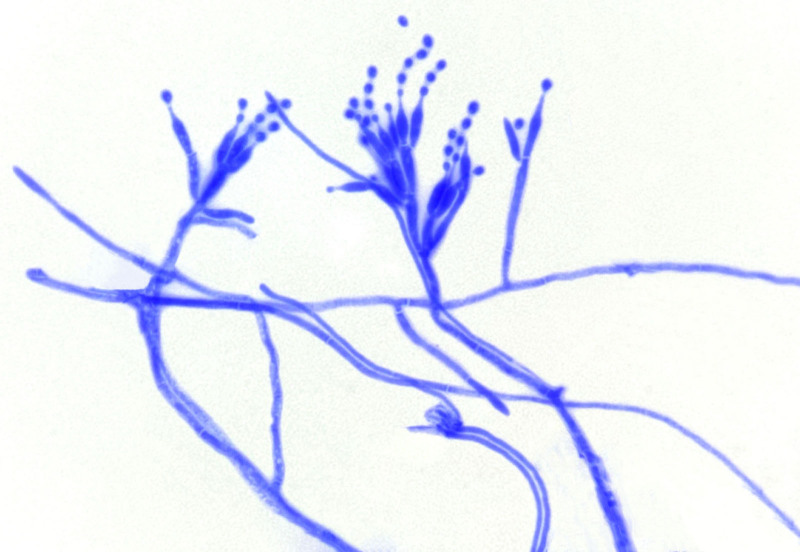
Laboratory findings under the microscope (400×): lactophenol cotton blue staining of the peripheral blood culture.

A man in his 40s presented to the Infectious Diseases Department II with persistent fever and progressive cutaneous desquamation, having been diagnosed with HIV 2 months earlier. At the time, he exhibited lymphadenopathy and dermatitis. A lymph node puncture revealed an infection with TM, and he was subsequently treated for atopic dermatitis. Two days prior to admission, his rash worsened, accompanied by increased peeling and keratinization, along with notable fever(maximum temperature 38.0℃), chest tightness, and shortness of breath, physical examination revealed extensive hyperkeratotic crusting resembling oyster shells, widespread desquamation, and multiple dark red macules and patches, some merging into larger plaques (Fig. 1), severe erosions were particularly evident in the perianal and scrotal regions. Laboratory tests showed a white blood cell count of 2.32 × 10^9^/L, with 81.5% neutrophils and 0.5% eosinophils, a procalcitonin level of 0.738 pg/mL (reference range: 0–500 pg/mL), C-reactive protein of 148.61 mg/L (reference range: 0–6 mg/L), albumin of 26.6 g/L, and prealbumin of 27 mg/L. His immunoglobulin E (IgE) level was markedly elevated at 1257.20 IU/mL, with a cluster of differentiation (CD) 4 count of just 3 cells/μL. The HIV RNA viral load measured 6.01 × 10^4^ IU/mL. The test results of galactomannan and 1, 3-β-d-glucan were 4.98 (reference range: 0–0.5) and 192.07 pg/mL (reference range: 0–60 pg/mL) respectively. Microscopy of skin scrapings showed *S scabiei* mites and eggs (Fig. 2), alongside dense eosinophilic infiltrates, confirming a diagnosis of Norwegian scabies. In response to the skin infection, meropenem was administered as part of the anti-infection regimen. In addition, Gram staining showed numerous yeast-like organisms after a 24 hours blood culture (Fig. 3). Lactophenol cotton blue staining also confirmed numerous yeast-like organisms for the peripheral blood culture (Fig. 4). After confirming the diagnosis of TM, voriconazole antifungal therapy was continued alongside ongoing antiretroviral therapy. The treatment focused on exterminating the mites, alleviating itching, and preventing potential complications. The patient made a full recovery and was discharged from the hospital following treatment with topical sulfur ointment, oral ivermectin, and oral voriconazole. Symptomatic supportive care included the administration of methylprednisolone for its anti-allergic effects and immunoglobulin therapy for additional support. Complete regression was confirmed after 1 month of treatment.

## 3. Discussion

Norwegian scabies, also referred to as crusted scabies, was first identified by Boeck and Danielssen in 1848 among leprosy patients in Norway. This rare clinical subtype of scabies is highly contagious and primarily affects immunocompromised individuals, including those infected with HIV, disabled patients, and the elderly.^[5]^ Its hallmark clinical manifestations include atypical skin lesions such as systemic erythematous scaling, crusting, or hyperkeratosis, often accompanied by eosinophilia and systemic lymphadenopathy. In immunocompromised patients, clinical presentations tend to be atypical, leading to frequent misdiagnosis as atopic dermatitis or psoriasis. A retrospective study in the United States found that 45% of scabies patients had been misdiagnosed.^[6]^ Although the exact percentage of misdiagnosis of scabies in HIV patients is unknown, the likelihood of misdiagnosis is high due to atypical presentations and overlap with other skin diseases.^[7,8]^ Insufficient awareness among clinicians regarding scabies and inappropriate treatments – such as the application of topical corticosteroids – can result in atypical clinical manifestations and delayed diagnoses. In immunodeficient patients, such as those with HIV, an inadequate immune response can facilitate the rapid progression of scabies to Norwegian scabies, exacerbating the condition.^[3,9]^

Delayed diagnosis can lead to various complications, including secondary impetigo, cellulitis, and sepsis, with mortality rates reaching as high as 50%. Furthermore, the high infectiousness of crusted scabies poses a risk for potential outbreaks.^[2,3,10]^ The World Health Organization classifies scabies as a neglected tropical disease, underscoring the frequent underestimation of its diagnosis and treatment.^[11]^

The case presented here involves an HIV patient who was severely immunosuppressed (with extremely low CD4⁺ T cell counts) and coinfected with TM. Initially, the patient’s skin lesions were atypical and misdiagnosed as atopic dermatitis, which contributed to disease progression. Microscopic examination of skin scrapings revealed the presence of scabies mites and eggs, leading to the final diagnosis of Norwegian scabies. The microscopic assessment of mites or eggs in skin scrapings serves as a straightforward and effective method for etiological examination. Scabies infections typically coincide with elevated IgE levels, which promote the recruitment of eosinophils to the skin. In this case, the patient’s IgE level was markedly elevated at 1257.20 IU/mL, with clusters of eosinophils observed in the skin scrapings. Research indicates that IgE levels and peripheral eosinophil counts in patients with Norwegian scabies correlate with the severity of skin reactions.^[12,13]^ Interestingly, the patient’s peripheral blood eosinophil count was not elevated, which may be attributed to CD4⁺ T cell exhaustion resulting from HIV infection, thereby weakening the Th2 immune response.^[1,14]^ Additionally, bone marrow suppression could impair the differentiation of hematopoietic progenitor cells, leading to reduced eosinophil production.

TM is an opportunistic fungal infection predominantly affecting HIV-infected individuals in tropical and subtropical regions of Asia.^[15,16]^ Disseminated infections can involve multiple organ systems, manifesting as fever, anemia, weight loss, fatigue, hepatosplenomegaly, lymphadenopathy, cough, sputum production, and gastrointestinal discomfort. Some patients may also exhibit central nervous system involvement.^[15,17,18]^ The recurrence and mortality rates associated with disseminated TM infection are alarmingly high; recurrences can occur even years later, with mortality rates ranging from 10% to 30%. If not promptly diagnosed and treated with antifungal therapy, the risk of death can escalate to 50%.^[4,19,20]^

In this case, the early atypical skin lesions may have resulted from the coinfection with scabies and TM, where the TM infection further exacerbated immunosuppression, facilitating scabies proliferation and creating a vicious cycle that obscured the typical manifestations of scabies. The concomitant presence of scabies and TM aggravated skin symptoms and complicated the diagnostic process. Therefore, in the context of this complex mixed infection, timely diagnosis and intervention are essential and warrant serious attention in clinical practice.

## 4. Conclusion

In conclusion, regular microscopy of skin scrapings should be primarily recommended for HIV-positive patients with unexplained dermatitis. The immunocompromised state, particularly in HIV disease, critically impairs the host’s capacity to regulate mite proliferation. Consequently, persistent papulosquamous eruptions in this population demand immediate consideration of Norwegian scabies to avert diagnostic oversight and subsequent infectious complications. Microscopic examination of skin scrapings for pathogenic examination is a simple and feasible procedure. It allows for the identification of scabies mites and other skin pathogens, aiding in accurate diagnosis and timely treatment.

## Acknowledgments

We thank all study participants and staff of all participating sites.

## Author contributions

**Research design:** Aifang Xu, Jing Wu.

**Physical examination and data collection:** Xiaojin Ding.

**Microscopic examination and data collection:** Wenyan Yu, Kenv Pan.

**Writing – original draft:** Yan Zhang.

**Writing – review & editing:** Aifang Xu, Yan Zhang.

## References

[1] Bergamin G Hudson J Currie BJ Mounsey KE. A systematic review of immunosuppressive risk factors and comorbidities associated with the development of crusted scabies. Int J Infect Dis. 2024;143:107036. 38570134 10.1016/j.ijid.2024.107036

[2] Hasan T Krause VL James C Currie BJ. Crusted scabies; a 2-year prospective study from the Northern Territory of Australia. PLoS NeglTrop Dis. 2020;14:e0008994. 10.1371/journal.pntd.0008994 PMC7781478 33338053

[3] Niode NJ Adji A Gazpers S. Crusted scabies, a neglected tropical disease: case series and literature review. Infect Dis Rep. 2022;14:479–91. 35735761 10.3390/idr14030051 PMC9223105

[4] Wang F Han R Chen S. An overlooked and underrated endemic mycosis-*Talaromycosis* and the pathogenic fungus *Talaromyces marneffei*. Clin Microbiol Rev. 2023;36:e0005122. 36648228 10.1128/cmr.00051-22 PMC10035316

[5] Karthikeyan K. Crusted scabies. Indian J Dermatol Venereol Leprol. 2009;75:340–7. 19584457 10.4103/0378-6323.53128

[6] Anderson KL Strowd LC. Epidemiology, diagnosis, and treatment of scabies in a dermatology office. J Am Board Fam Med. 2017;30:78–84. 28062820 10.3122/jabfm.2017.01.160190

[7] Pakanati K Jagota D Ladogana M. Norwegian scabies in HIV/AIDS. Proc (Bayl Univ Med Cent). 2022;35:346–7. 35518806 10.1080/08998280.2022.2028705 PMC9037398

[8] Yari N Malone CH Rivas A. Misdiagnosed crusted scabies in an AIDS patient leads to hyperinfestation. Cutis. 2017;99:202–4. 28398415

[9] Thomas C Coates SJ Engelman D Chosidow O Chang AY. Ectoparasites: scabies. J Am Acad Dermatol. 2020;82:533–48. 31310840 10.1016/j.jaad.2019.05.109

[10] Fernando DD Mounsey KE Bernigaud C. Scabies. Nat Rev Dis Primers. 2024;10:74. 39362885 10.1038/s41572-024-00552-8

[11] Engelman D Marks M Steer AC. A framework for scabies control. PLoS NeglTrop Dis. 2021;15:e0009661. 10.1371/journal.pntd.0009661 PMC8412357 34473725

[12] Liu X Walton SF Murray HC. Crusted Scabies is associated with increased IL-17 secretion by skin T cells. Parasite Immunol. 2014;36:594–604. 25040151 10.1111/pim.12129

[13] Mounsey KE Murray HC Bielefeldt-Ohmann H. Prospective study in a porcine model of *Sarcoptes scabiei* indicates the association of Th2 and Th17 pathways with the clinical severity of Scabies. PLoS NeglTrop Dis. 2015;9:e0003498. 10.1371/journal.pntd.0003498 PMC4346266 25730203

[14] Walton SF Beroukas D Roberts-Thomson P Currie BJ. New insights into disease pathogenesis in crusted (Norwegian) scabies: the skin immune response in crusted scabies. Br J Dermatol. 2008;158:1247–55. 18422789 10.1111/j.1365-2133.2008.08541.x

[15] Cao C Xi L Chaturvedi V. Talaromycosis (Penicilliosis) due to *Talaromyces (Penicillium*) *marneffei*: insights into the clinical trends of a major fungal disease 60 years after the discovery of the pathogen. Mycopathologia. 2019;184:709–20. 31811603 10.1007/s11046-019-00410-2

[16] Pruksaphon K Nosanchuk JD Ratanabanangkoon K Youngchim S. *Talaromyces marneffei* infection: virulence, intracellular lifestyle and host defense mechanisms. J Fungi (Basel). 2022;8:200. 35205954 10.3390/jof8020200 PMC8880324

[17] Chan JF Lau SK Yuen KY Woo PCY. *Talaromyces* (*Penicillium*) *marneffei* infection in non-HIV-infected patients. Emerg Microbes Infect. 2016;5:e19. 26956447 10.1038/emi.2016.18 PMC4820671

[18] Liu X Xing H Lin J. Coexisting of primary central nervous system lymphoma and *Talaromyces marneffei* brain abscess in an AIDS patient, a case report and review of the literature. Infect Drug Resist. 2024;17:709–18. 38410795 10.2147/IDR.S432697 PMC10896102

[19] Chastain DB Henao-Martínez AF Franco-Paredes C. Opportunistic invasive mycoses in AIDS: cryptococcosis, histoplasmosis, coccidiodomycosis, and *Talaromycosis*. Curr Infect Dis Rep. 2017;19:36. 28831671 10.1007/s11908-017-0592-7

[20] Chen L Yu L Wu Y Ming W-K Huang Z Liu S. A 52-year-old man with an 11-month history of fever, cough, chest pain, pleural effusion, and left lung atelectasis. Chest. 2020;158:e153–7. 33036111 10.1016/j.chest.2020.05.546

